# Systematic Review: Maternal Risk Factors, Socioeconomic Influences, Neonatal Biomarkers and Management of Early-Onset Sepsis in Late Preterm and Term Newborns—A Focus on European and Eastern European Contexts

**DOI:** 10.3390/life15020292

**Published:** 2025-02-13

**Authors:** Anca Vulcănescu, Mirela-Anișoara Siminel, Sorin-Nicolae Dinescu, Mihail-Virgil Boldeanu, Anda-Lorena Dijmărescu, Maria-Magdalena Manolea, Constantin-Cristian Văduva

**Affiliations:** 1 “Filantropia” Clinical Municipal Hospital, 200143 Craiova, Romania; anca.vulcanescu@umfcv.ro (A.V.); mihail.boldeanu@umfcv.ro (M.-V.B.); lorena.dijmarescu@umfcv.ro (A.-L.D.); magdalena.manolea@umfcv.ro (M.-M.M.); cristian.vaduva@umfcv.ro (C.-C.V.); 2 University of Medicine and Pharmacy of Craiova, 200349 Craiova, Romania; 3 Department of Neonatology, University of Medicine and Pharmacy of Craiova, 200349 Craiova, Romania; 4 Department of Epidemiology, University of Medicine and Pharmacy of Craiova, 200349 Craiova, Romania; sorin.dinescu@umfcv.ro; 5 Clinical Emergency County Hospital, 200642 Craiova, Romania; 6 Department of Immunology, University of Medicine and Pharmacy of Craiova, 200349 Craiova, Romania; 7 Department of Obstetrics and Gynecology, University of Medicine and Pharmacy of Craiova, 200349 Craiova, Romania

**Keywords:** early-onset sepsis, maternal risk factors, Eastern Europe

## Abstract

Early-onset sepsis (EOS) remains a major cause of neonatal morbidity and mortality worldwide, with significant differences in the incidence and outcome of the disease in Europe. Eastern European countries face particular challenges due to differences in access to healthcare, diagnostic facilities, and prevention strategies. This review summarizes the results of recent research to provide insights into maternal risk factors, regional inequalities in access to healthcare, diagnostic biomarkers, pathogen patterns, and treatment protocols for EOS. This review also examines how healthcare infrastructure and socioeconomic factors influence EOS outcomes in Eastern Europe. Introduction: Early-onset sepsis (EOS) presents a significant health challenge for newborns, characterized by sepsis occurring within the first 72 h of life, primarily caused by the vertical transmission of pathogens from mother to child. Despite advancements in medical care, EOS remains particularly burdensome in resource-poor settings, especially in Eastern Europe, where disparities in healthcare access and maternal health are pronounced. This systematic review aims to provide insights into maternal risk factors, regional inequalities in healthcare access, diagnostic biomarkers, pathogen patterns, and treatment protocols for EOS. Background/Objectives: EOS is increasingly recognized as a public health issue, with outcomes significantly influenced by maternal health, socioeconomic status, and healthcare infrastructure. The review seeks to summarize the existing literature on EOS, particularly focusing on differences between high-income Western and low-resource Eastern European countries. The influence of maternal access to antenatal care, pathogen prevalence, and antibiotic resistance on EOS outcomes across regions will also be examined. Methods: To achieve the review’s objectives, a comprehensive search was conducted across multiple databases including PubMed, Google Scholar, ScienceDirect, and Scopus, adhering to PRISMA guidelines for systematic reviews. The inclusion criteria encompassed studies published within the last 20 years (January 2004–August 2024) that addressed EOS in late preterm or term infants, emphasizing maternal health, risk factors, diagnostic approaches, and treatment protocols pertinent to European populations. Exclusion criteria included non-English publications and studies lacking a focus on maternal and neonatal health. A total of 29 peer-reviewed articles meeting the specified criteria were ultimately included in the analysis. Results: The findings highlight significant regional disparities in EOS management between Western and Eastern Europe. Key issues include maternal risk factors, socioeconomic barriers to healthcare, diagnostic biomarkers, and pathogen resistance trends. Limited access to prenatal screenings and healthcare infrastructure in Eastern European countries, especially in rural regions in Romania, exacerbate the challenges faced by expectant mothers. Financial burdens, such as high out-of-pocket expenses, were shown to further restrict access to necessary maternal care. Conclusions: This systematic review emphasizes the urgent need for targeted investments in maternal healthcare infrastructure in Eastern Europe to mitigate the impacts of EOS. Enhanced screening programs, standardized surveillance systems, and ensuring equitable health policies are essential to improving neonatal outcomes. Additionally, tailored education and awareness campaigns for disadvantaged groups and comprehensive health policy reforms, including universal antenatal care and Group B Streptococcus (GBS), are essential to bridging healthcare gaps.

## 1. Introduction

Early-onset sepsis (EOS) is defined as sepsis that occurs within the first 72 h of life and is primarily caused by the vertical transmission of pathogens from the mother to the newborn during labor or delivery. Despite advances in healthcare, EOS remains a significant burden for newborns, particularly in resource-poor settings. Factors such as maternal access to adequate antenatal care, pathogen prevalence, diagnostic technologies, and antibiotic resistance contribute to the variation in EOS outcomes across regions.

In Europe, there are major differences between high-income Western countries and Eastern European countries with comparatively low resources in terms of prevention, diagnosis, and management of EOS [1,2,3,4,5,6,7,8,9,10,11,12,13,14].

## 2. Methods

The aim of this review was to summarize the available data on early-onset sepsis in newborns, focusing on regional differences in Europe, particularly regarding maternal health.

### 2.1. Search Strategy

**Figure 1:**
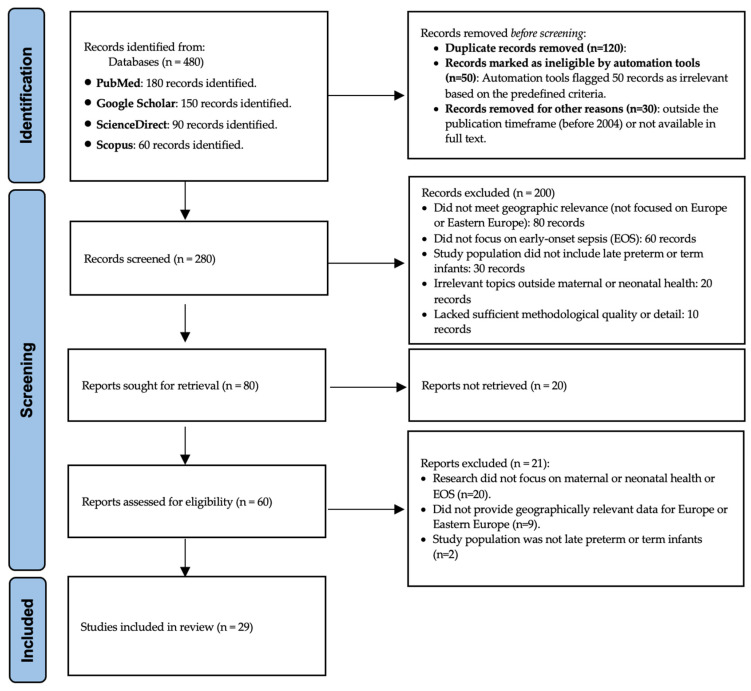
Prisma flow chart.

This systematic review followed the PRISMA (Preferred Reporting Items for Systematic Reviews and Meta-Analyses) guidelines to ensure a structured and transparent approach to literature search, data collection, and synthesis. The literature search (Figure 1) was conducted in four major electronic databases—PubMed, Google Scholar, ScienceDirect, and Scopus—by two of the authors (A.V. and M.S.; C.V. was the third reviewer, responsible for the final decision). Studies were searched and retrieved between September and October 2024, based on a predefined search strategy that included a combination of specific keywords and Boolean operators to refine and filter the results. Keywords used in the search included “Early-Onset Sepsis”, “neonatal sepsis”, “Group B Streptococcus”, “maternal healthcare”, and “Europe”, combined using Boolean operators such as AND, OR, and NOT. For example, search strings included combinations like “Early-Onset Sepsis” AND “Group B Streptococcus” AND “Europe”.

This review focused on articles published in the last 20 years (January 2004–August 2024) to capture the most relevant and up-to-date research. The extensive search strategy can be found in Supplementary Material S1.

### 2.2. Inclusion and Exclusion Criteria

Inclusion Criteria:

- Articles published between January 2004 and August 2024.
- Peer-reviewed studies (comprising original research articles, reviews, meta-analyses, and guidelines) focusing on early-onset sepsis (EOS) in late preterm or term infants.
- Studies investigating maternal or neonatal risk factors, diagnostic biomarkers or methods, prevention strategies, treatment protocols, or antibiotic resistance trends related to EOS.
- Studies providing data or evidence specific to European and Eastern European populations.
- Full-text articles available in English.

Exclusion Criteria:

- Articles not available in full-text format.
- Articles that were conference abstracts, commentaries, or single-case reports.
- Studies published in non-English languages.
- Research that did not directly relate to maternal or neonatal health or EOS.
- Studies focusing on populations outside the geographical scope of Europe and Eastern Europe.

### 2.3. Data Extraction

Data from eligible studies were extracted and included the following study parameters: author(s), year of publication, study design, key outcomes related to EOS, such as identified risk factors, pathogen prevalence, diagnostic approaches, biomarkers, treatment protocols, and regional disparities in healthcare access, geographic focus, and relevance to European or Eastern European contexts.

Risk of Bias Assessment was conducted by two of the authors (A.V. and M.S.). To evaluate the quality of included studies, we used general research guidelines, focusing on factors such as study design, sample size, outcome reporting, funding bias, and conflicts of interest. Extended results can be found in Supplementary Material S2.

Effect measures were used such as relative risk (used to compare the likelihood of EOS in different maternal risk groups, comparing EOS risk in mothers who received GBS screening and prophylaxis versus those who did not) and odds ratios (applied for outcomes such as the presence of GBS colonization or EOS occurrence in neonates born to mothers with premature rupture of membranes—PROM), and the mean difference was used for continuous variables like biomarkers (e.g., CRP levels).

Subgroup analysis was performed to evaluate differences between high-income and low-income European countries or other subgroups (e.g., rural versus urban settings) and descriptive statistics were used to summarize study characteristics, such as sample size, maternal demographics, and diagnostic approaches, using frequencies and percentages for categorical variables and means for continuous variables.

Regarding the statistical software, we used Microsoft Excel (Version 16.93.1 (25011917)) and macOS numbers (version 14.3 (7042.0.76)) (used to organize raw data and generate simple summary statistics). No specialized bias assessment software was directly used.

### 2.4. Results

The search yielded a total of 29 articles that met the inclusion criteria. These articles were analyzed to identify major trends and knowledge gaps related to EOS. Topics addressed in the selected studies include maternal risk factors, socioeconomic barriers, the role of prenatal screening, diagnostic advancements in EOS, and pathogen patterns with antibiotic resistance trends (Table 1 and Table 2).

To maintain the focus and relevance of this review, we excluded studies that did not align with the objectives or population scope. For example, Fleischmann et al. (2021) [30] also focused on late-onset sepsis, while Murthy et al. (2018) [31] examined socioeconomic factors in India, a region outside the geographic scope of this analysis. These exclusions ensured that the included studies provided direct insights into maternal risk factors and socioeconomic disparities impacting early-onset sepsis within European contexts.

## 3. Review Findings

### 3.1. Incidence of EOS in the European Context

Western Europe benefits from robust screening and prophylaxis programs that lead to a lower incidence of EOS. Schrag et al. (2016) estimated EOS rates in high-income European countries to be around 0.3–0.5 cases per 1000 live births, primarily due to the widespread introduction of screening for Group B Streptococcus (GBS) [1].

In contrast, Eastern European countries have a higher incidence of EOS, as reported by Barcaite et al. (2008) and Miteniece (2021), due to gaps in access to maternal healthcare and inconsistent implementation of screening protocols [5,12,32,33].

The estimated incidence of EOS in Romania is between 0.5 and 1 per 1000 live births in full-term infants and up to 10 per 1000 live births in late preterm infants [11,33].

The high neonatal mortality rate in Romania (defined as death within the first 28 days of life) is influenced by EOS, as Tarca et al. (2016) pointed out [34]. According to WHO and UNICEF, Romania has one of the highest neonatal mortality rates in the European Union (EU). According to recent reports, the neonatal mortality rate is 3.1 deaths per 1000 live births [6,35]. In comparison, the average neonatal mortality rate in the EU is around 2.7 deaths per 1000 live births. The prevalence of infections and prematurity have been identified as the main causes of neonatal sepsis and mortality in Romania.

### 3.2. Socioeconomic Barriers to Maternal Healthcare Access

Economic poverty and financial obstacles: Economic constraints are a major barrier to accessing routine antenatal care, especially in resource-poor settings. High costs of healthcare services discourage pregnant women from seeking timely and frequent maternity care [3,26]. This is particularly pronounced in Eastern European countries such as Romania, where poverty further limits access to preventive care and early detection of risk factors for EOS [9,36].

Inefficient healthcare systems: Poor healthcare infrastructure and inadequate provision of maternal health services in low-resource settings contribute significantly to inadequate antenatal care. Delays in screening for maternal infections (e.g., testing for Group B Streptococcus [GBS]) are common in health systems that lack standardized protocols or sufficient funding for universal screening [3,9]. Limited diagnostic capacity in such settings also impacts the ability to detect and prevent maternal illnesses associated with neonatal infections [18,21].

Geographical disparities and urban–rural divide: Rural communities often face severe geographic barriers to accessing maternal and obstetric care. These areas tend to have fewer healthcare providers, poorer accessibility to hospitals, and poorly developed transportation networks, making it difficult for pregnant women to reach important services such as prenatal check-ups [20,28,36]. This urban–rural divide is particularly problematic in countries with significant regional disparities, such as Romania and Eastern Europe [9]. According to Eurostat, around 20% of pregnant women in rural areas of Romania do not have adequate access to antenatal services [37].

Inconsistent prenatal screening programs: The lack of universal maternal screening programs, especially for infections such as GBS, contributes significantly to the risk of EOS in newborns in countries with fragmented health systems. For example, disparities in GBS screening and intrapartum antibiotic prophylaxis have been found in Europe due to differences in healthcare funding and policies [1,27].

Outdated policies and inefficient administration: gaps in policies, including the lack of universal maternal health coverage or funding for maternal care programs, result in missed opportunities for preventive care [9]. In many countries, there is a lack of sufficient guidelines or implementation of antenatal care recommendations, leading to inequitable healthcare [2].

Cultural and educational factors: Socioeconomic barriers often intersect with cultural and educational differences. Inadequate maternal health literacy due to insufficient education limits awareness of the importance of antenatal care, GBS screening, and timely access to healthcare. Cultural norms in certain regions may also discourage women from seeking formal healthcare providers, especially in underserved populations [28,36].

Migration and vulnerable populations: Refugees, asylum seekers, and marginalized groups often face additional socioeconomic barriers, including language barriers, limited knowledge of healthcare systems, and limited access to health insurance or public services. Records show that approximately 1400 refugee mothers and their children have gained access to some form of maternal healthcare through partnerships with UNICEF in Romania [38]. Roma women make up a large proportion of underrepresented and underserved mothers in Romania [39]. These populations are at increased risk of infections and complications that increase the risk of EOS in newborns [3].

The shortage of healthcare providers and laborers is a systematic problem in resource-limited areas, especially in rural areas. The lack of obstetricians, midwives, and trained health workers contributes to significant delays in basic maternal care [3,9,26].

Underlying social inequalities, including gender inequalities in access to healthcare, affect maternal health outcomes. In patriarchal societies, women’s limited access to decision-making and health resources hinders their ability and autonomy to access necessary care during pregnancy [9,28].

Global health disparities: Systematic inequalities in the global distribution of health resources lead to cascading risks for maternal and newborn outcomes. High-income countries with universal health coverage and widespread GBS screening have significantly lower incidence rates of EOS than regions with limited resource allocation [1,27].

### 3.3. Maternal Risk Factors for EOS

Maternal risk factors play a central role in the development of early-onset sepsis (EOS), which manifests within the first 72 h of life through the vertical transmission of pathogens from mother to newborn during pregnancy or birth.

- Colonization with Group B Streptococcus (GBS) remains one of the most important risk factors for EOS. GBS from the maternal genital or rectal flora is transmitted to the newborn during birth if left untreated [7,23,24]. In regions with inconsistent GBS screening measures or a lack of intrapartum antibiotic prophylaxis (IAP), the risk of GBS-associated EOS is higher [8,27].
- Prolonged rupture of membranes (PROM), defined as the rupture of amniotic membranes >18 h before birth, is strongly associated with neonatal infections [16,28,40]. PROM increases the risk of ascending bacterial infections from the genital tract to the amniotic sac and exposes the newborn to sepsis-causing pathogens.
- Maternal fever during labor (≥38 °C) is a clinical indicator of possible intraamniotic or systemic infection. It contributes to neonatal exposure to pathogens and an increased likelihood of sepsis, especially in the absence of adequate maternal screening or prophylaxis [4,40].
- Chorioamnionitis, the inflammation of the fetal membranes and amniotic fluid due to bacterial infection, is another critical risk factor. This condition, often associated with PROM and prolonged labor, increases the neonate’s exposure to pathogenic microorganisms [2,11,24].
- Premature labor and premature birth not only lead to immaturity of the newborn but also expose premature infants to a higher risk of infection. The Lancet study by Kwatra et al. (2016) emphasizes that infants born before full term are at increased risk due to their immature immune system, limited transplacental antibody transfer, and other physiological vulnerabilities [23]. Prolonged exposure to invasive procedures further increases the likelihood of EOS for maternal infections during preterm birth [18,22].
- Prolonged labor increases the risk of maternal and neonatal infections as the birth canal comes into contact with pathogens for longer. It increases the potential for vertical transmission, especially in GBS-positive or febrile mothers [14,15].
- Prenatal maternal genitourinary tract infections, including urinary tract infections (UTIs) and sexually transmitted infections (STIs), can lead to ascending infections resulting in preterm labor and EOS [17,28].
- The lack of routine maternal screening and preventive measures contributes to the underdiagnosis of maternal infections, including GBS colonization. This is particularly significant in low-resource settings and leads to missed opportunities for prophylaxis and EOS prevention [1,9,32].
- Maternal comorbidities, particularly hypertensive disorders (e.g., pre-eclampsia) and gestational diabetes, are common in Romania and contribute to adverse pregnancy outcomes. These conditions increase the risk of early-onset sepsis (EOS) due to complications such as preterm delivery, prolonged rupture of the membranes, and the need for invasive obstetric interventions, which can facilitate neonatal exposure to pathogenic bacteria [24,41]. Additionally, surgical treatments for pregnancy-related issues, including ovarian endometriomas, ovarian cyst torsion, and even appendicitis, further complicate maternal management. While necessary, these procedures may lead to increased rates of intrauterine infection, preterm birth, and disruptions in maternal microbiota, all of which are known risk factors for EOS in neonates. Vaduva et al. (2023) highlighted the long-term reproductive health implications of such surgeries, indicating how they can affect future fertility [42]. While necessary, surgeries carry risks that may worsen existing maternal comorbidities, potentially leading to postoperative complications that increase morbidity for mothers and infants. Effective pain management during these procedures is crucial, as highlighted by Costea et al. (2022), to reduce complications and prioritize maternal well-being. Understanding the relationship between maternal comorbidities and surgical interventions is vital for optimizing prenatal care and enhancing health outcomes for mothers and newborns, considering the potential for postoperative infections or inflammatory responses that can contribute to neonatal sepsis risk [43].
- Socioeconomic factors and inequitable healthcare contribute indirectly to higher EOS risk by limiting access to quality prenatal care, routine GBS screening, and intrapartum interventions. Regions with greater poverty and limited health infrastructure, such as parts of Eastern Europe, are disproportionately affected [1,3,26,44].
- Inadequate intrapartum antibiotic prophylaxis (IAP) for GBS-positive mothers significantly increases the neonatal EOS risk. This is often due to either a lack of GBS screening or delays in initiating antibiotic treatment during labor [16,27].
- Multiple cesarean deliveries and placental disorders such as placenta-accreta spectrum increase the risk of preterm birth, bleeding, and invasive procedures that put the newborn at risk of infection [21].
- Multidrug-resistant maternal infections can complicate treatment and increase neonatal exposure to resistant bacteria during labor and delivery [4,10].

### 3.4. Diagnostic Biomarkers and EOS Risk Stratification

The EOS diagnostic approaches in Eastern Europe are very different. Some countries, such as Poland and Hungary, adopt local versions of international guidelines such as those of NICE (National Institute for Health and Care Excellence), while others, including Romania and Bulgaria, rely on less standardized protocols or medical judgment [3,18].

- Biomarkers in EOS Diagnosis

Neonatal biomarkers serve as valuable tools for the detection of EOS by assessing inflammatory reactions, bacterial infections, or tissue damage in newborns. In several studies, the following biomarkers have been reported to be effective for EOS diagnostics:

C-reactive protein (CRP): CRP is one of the most used biomarkers for neonatal sepsis, measured in neonatal blood samples. Elevated CRP levels correlate with the severity of the infection and provide information about the inflammation [13,14]. However, CRP alone has limitations due to its delayed rise (within 6–12 h after the onset of infection), which reduces its sensitivity in the early phase of EOS [14].

Procalcitonin (PCT): PCT, measured in neonatal blood, is an inflammatory marker increasingly recognized for its usefulness in the diagnosis of EOS. It has a faster onset than CRP and is specific for bacterial infections, with lower levels in viral infections. Dynamic changes in PCT levels can help to rule out or confirm EOS at an early stage and inform decisions to limit unnecessary antibiotic use [13,14].

White blood cell (WBC) count and ratios: Neonatal blood samples are analyzed for absolute neutrophil counts and the ratio of immature to total white blood cells, which serve as complementary diagnostic tools. However, their low specificity and susceptibility to maternal or perinatal factors limit their reliability in EOS diagnostics [7,25].

Neutrophil-to-lymphocyte ratio (NLR): NLR, derived from neonatal blood tests, has been identified as a promising biomarker that has high diagnostic accuracy for EOS when combined with clinical parameters [22].

Cytokines and chemokines: Inflammatory markers such as IL-6 and IL-8, measured in neonatal blood, have been investigated for their potential use in EOS diagnostics. These cytokines rise earlier than CRP and PCT but are not routinely used due to the complexity and cost of the tests [14]. Gene polymorphisms in Toll-like receptors (TLR-2 and TLR-4) also showed correlations with EOS risk, highlighting the need for genetic screening in high-risk newborns [45].

Microbiological cultures: Blood cultures remain the gold standard for EOS diagnosis, although delayed results and the low volume of neonatal blood samples reduce sensitivity [4,13]. Pathogen-specific cultures are crucial for the confirmation of EOS and identification of antibiotic resistance but may be limited by delays in resource-limited regions [4,10].

Zonda et al. (2019) investigated the use of Endocan, a biomarker measured in neonatal blood, which has significant potential for the diagnosis of EOS in neonates [33]. In addition, D-dimer levels were analyzed in neonatal blood samples in a single-center study to determine their diagnostic relevance, which proved useful in the clinical setting [46]. Hincu et al. (2024) provided an overview of common and emerging neonatal blood biomarkers, noting that while conventional markers are widely used, newer markers such as presepsin and procalcitonin offer promising results for early detection [22].

b. Advances in Risk Stratification Tools

Risk-based EOS stratification models allow healthcare providers to identify newborns who are at higher or lower risk of developing sepsis, allowing for more precise and tailored management. These strategies take into account biomarker data, maternal risk factors, and the clinical situation.

Kaiser Permanente Neonatal Early-Onset Sepsis Calculator: The EOS risk calculator is a widely validated tool developed to integrate maternal risk factors (e.g., maternal fever, GBS status, PROM) with the clinical presentation and laboratory findings of the newborn [11,12]. This real-time tool quantifies the likelihood of sepsis and helps to decide whether to initiate empirical antibiotics, perform additional tests, or simply observe the newborn. The challenges in implementing this tool include the variability in thresholds and availability of complete maternal data [12].

Combined Biomarker Models: Combined biomarker analyses improve the sensitivity and specificity of EOS diagnostics. For example, CRP and PCT, which are measured together at several intervals, provide a more accurate risk classification than each individual biomarker [13,14].

Clinical Risk-Scoring Systems: Risk-scoring systems stratify newborns based on maternal, perinatal, and neonatal variables such as maternal GBS status, gestational age, and clinical symptoms. These clinical models guide stratification but may have limited sensitivity in detecting milder forms of EOS [1,28]. Studies from Romania and Bulgaria highlighted adaptations of risk-based approaches, which often rely on additional clinical assessments in the absence of complete diagnostic tools [18,22].

c. Challenges in Biomarker Use and Risk Stratification

Despite progress, there are still problems with the general introduction of biomarkers and risk stratification tools:

Cost and Resource Constraints: Advanced biomarkers such as procalcitonin and cytokines are expensive and may not be available in resource-poor settings [3,44].

False Positives/False Negatives: Many biomarkers lack absolute specificity, leading to false positive or negative results, which can result in unnecessary treatment or missed cases [14].

Delayed Culture Results: As microbiological culture results are often delayed, many EOS cases are empirically treated with antibiotics before confirmation, which increases the risk of overuse of antibiotics and resistance [4,10].

Variability in Hospital Protocols: The lack of standardized approaches to risk stratification in the various healthcare systems leads to the inconsistent adoption of biomarkers and calculation methods [1,12].

d. Biomarkers and Stratification as Tools for Antimicrobial Stewardship

Innovative strategies that combine biomarker evaluation with risk stratification tools support antibiotic stewardship by reducing unnecessary empirical antibiotic therapy in low-risk neonates through risk-adaptive management, identifying high-risk newborns in need of immediate and aggressive treatment, improving outcomes and reducing mortality, and adjusting antibiotic duration based on serial PCT measurements, which have been shown to minimize the duration of unnecessary antibiotic exposure [11,13,14,15,28].

### 3.5. Pathogens and Resistance Trends

- Causative Pathogens

The most common pathogens of EOS originate primarily from the maternal flora, which is transmitted during birth. Their prevalence varies by region, infection prevention practices, and healthcare facilities.

Group B Streptococcus (GBS): GBS remains the leading cause of EOS worldwide and in Europe if systematic screening and antibiotic prophylaxis are not carried out [1,23,24].

Gram-Negative Bacteria (e.g., *Escherichia coli*): *E. coli* is an important cause of EOS, especially in premature infants. It is associated with severe infections and higher mortality rates than GBS, especially in situations where early maternal screening is inadequate [4,13].

Other Pathogens: these include *Klebsiella* spp., *Enterobacter* spp., *Proteus* spp., and *Listeria monocytogenes*. Fungal pathogens such as *Candida* spp. are less common but are found in preterm infants in resource-intensive intensive care units [10,14].

Johansson Gudjónsdóttir et al. (2019) and Flidel-Rimon et al. (2022) reported a shift from Gram-positive to Gram-negative pathogens in European neonatal units, particularly in Eastern Europe [10,25]. This transition is attributed to increasing antibiotic resistance in Gram-negative organisms.

b. Antimicrobial Resistance Trends

Giannoni et al. (2018) highlighted worrying trends in antimicrobial resistance in neonatal sepsis pathogens, with Eastern European countries disproportionately affected due to the overuse of broad-spectrum antibiotics [4]. The continued emergence of antimicrobial resistance in EOS pathogens makes effective treatment difficult and is influenced by the overuse or misuse of antibiotics in both neonates and mothers.

GBS resistance: While penicillin remains the drug of choice for GBS, resistance to alternative treatments such as macrolides and clindamycin has occasionally been documented, limiting options for penicillin-allergic patients [23,27].

Gram-Negative Resistance: *E. coli* isolates are increasingly showing resistance to beta-lactam antibiotics, including ESBL-producing strains (extended-spectrum beta-lactamase), which makes treatment more difficult [4]. Resistance to aminoglycosides, which are frequently used in combination therapy, is also increasing in some regions [10].

Global disparities: Resistance to neonatal pathogens is more common in resource-poor settings where the overuse of broad-spectrum antibiotics is widespread and infection control practices are limited [1,24]. The rate of multidrug resistance (e.g., carbapenem-resistant *Klebsiella* spp. and multidrug-resistant *E. coli*) is higher in certain regions.

### 3.6. Prevention Strategies and Treatment Protocols

- Prevention Strategies

Prevention strategies focus on intrapartum care, maternal infection screening, and minimizing unnecessary interventions to reduce the transmission of pathogens.

GBS Screening and Intrapartum Antibiotic Prophylaxis (IAP): universal GBS screening and the administration of IAP to colonized mothers are the cornerstone of EOS prevention in high-income countries. This approach has significantly reduced the incidence of GBS-related EOS [1,27]. However, its global effectiveness is limited by inconsistent implementation in low-resource settings [7]. Berardi et al. (2017) reviewed European guidelines for GBS screening and intrapartum antibiotic prophylaxis (IAP) and found inconsistent implementation, particularly in Eastern Europe [27]. While countries such as the UK and France have introduced universal screening, Romania and other Eastern European countries rely on risk-based approaches.

Improved prenatal care: the detection and treatment of maternal infections, such as urinary tract infections and chorioamnionitis, during pregnancy reduce the risk of EOS [12,28].

Hygiene and infection control: adherence to universal precautions, including proper hand hygiene and aseptic birthing practices, is critical to minimize the introduction of external pathogens [4,23]. This is particularly important in settings where multiple births occur in resource-limited health facilities.

Antimicrobial Stewardship: reducing the unnecessary use of antibiotics in the postpartum period and in newborns is crucial to minimize the development of resistance. This includes limiting broad-spectrum antibiotic prophylaxis to cases with clearly identified risk factors [25].

Vaccination strategies: A maternal vaccine against GBS is under development and could represent a significant advance in reducing vertical transmission of this pathogen [23].

b. Treatment Protocols

EOS treatment protocols aim to strike a balance between effective pathogen eradication and minimizing unnecessary antibiotic exposure that contributes to the development of resistance.

Empiric Antibiotic Therapy.

The first empirical therapy for suspected EOS usually consists of:

- Ampicillin (to cover GBS and *Listeria monocytogenes*); and
- Gentamicin (to cover Gram-negative organisms) [5,40]. This combination is still effective in most regions, although local resistance patterns may require alternative therapies, such as the addition of cefotaxime for Gram-negative resistance [14,29].

Pathogen-Specific Adjustments: As soon as the results of the blood cultures are available, the therapy is tailored to the identified pathogen and its antimicrobial susceptibility profile [10,25].

Shortened Antibiotic Courses for Low-Risk Neonates: Procalcitonin (PCT) and serial measurements of C-reactive protein (CRP) allow clinicians to discontinue antibiotics early in neonates who are no longer considered at risk after negative cultures and clinical improvement [12,14].

Antibiotic stewardship: Hansen et al. (2016) documented differences in empirical antibiotic regimens, with Eastern European countries more likely to use broad-spectrum antibiotics, contributing to the resistance patterns observed by Flidel-Rimon et al. (2022) [10,28]. The guidelines recommend the use of broad-spectrum antibiotics and minimizing prolonged exposure to broad-spectrum agents unless clinically indicated [13,28].

## 4. Discussion

### 4.1. Disparities Between Western and Eastern Europe

Healthcare infrastructure, socioeconomic factors, and public health priorities have a significant impact on maternal and neonatal outcomes, including the incidence and treatment of early-onset sepsis (EOS). Significant differences exist between Western and Eastern Europe in terms of preventive practices, access to maternal healthcare, and screening.

Healthcare infrastructure and resource availability: Western Europe generally benefits from well-funded, universal healthcare systems with broad access to high-quality prenatal care. For example, in countries such as the UK, France, and Germany, routine screening for infections such as Group B Streptococcus (GBS) is standard [1,24,27]. In contrast, access to healthcare in Eastern Europe is hampered by underfunded public healthcare systems that place less emphasis on preventive healthcare [3,19,44].

Rates of GBS screening implementation: Universal GBS screening, followed by intrapartum antibiotic prophylaxis (IAP), is a cornerstone of antenatal care in Western Europe [47,48,49]. However, in Eastern European countries, GBS screening is inconsistently implemented due to funding limitations, a lack of standardized protocols, and inequalities in healthcare [3,7,44,50,51]. Limited access to antenatal care further exacerbates inequalities in the detection of maternal GBS colonization [52]. 

Socioeconomic and Regional Inequalities: poverty and regional inequalities in Eastern Europe lead to lower healthcare utilization and perinatal mortality rates that lag behind those in Western Europe [6,20,53,54]. Rural areas face significant barriers, including a lack of maternity services and fewer health professionals [18,21,52,55]. Although the cost of prenatal care in Romania is partially subsidized, it remains an obstacle for many families. The average expenditure for prenatal visits, including ultrasounds and necessary tests, is between 200 and 500 euros, with additional costs for specialized tests such as fetal echocardiograms when these are recommended. For those who are uninsured, the cost for the entire duration of prenatal care can be as high as 1500 EUR, making it difficult for low-income families to meet the recommended schedule [9].

Antimicrobial Practices: antibiotic stewardship programs are more widespread in Western Europe, where the rational use of empirical antibiotics in the treatment of EOS is emphasized [56,57]. Eastern European countries report more frequent overuse and inappropriate antibiotic regimens that contribute to greater antimicrobial resistance [4,10].

Economic barriers, inequalities in rural healthcare, and inconsistent implementation of guidelines exacerbate poor neonatal care outcomes in Eastern Europe [3,26].

### 4.2. Importance of Maternal Healthcare Access

Access to comprehensive maternal healthcare is critical to reducing the incidence of EOS and ensuring better neonatal outcomes. Gaps in access contribute directly to increased neonatal morbidity and mortality.

Timely Detection and Treatment of Maternal Infections: routine antenatal care and infection screening prevent neonatal infections by enabling the timely treatment of maternal conditions such as GBS colonization, chorioamnionitis, and urinary tract infections [12,15,48,58]. In regions with limited access to maternal healthcare, these conditions often go untreated, increasing the risk of EOS [18].

Impact on Neonatal Outcomes: comprehensive maternal care directly reduces neonatal complications such as EOS, premature birth, and low birth weight [59,60]. The Western European model of universal health coverage is an example of how equitable access can lead to better outcomes than in the underfunded systems of Eastern Europe [3,9].

Role of Education and Outreach Programs: improved maternal health care includes educating pregnant women about risk factors, signs of preterm labor, and the importance of routine check-ups. These strategies are underutilized in Eastern Europe, further widening the gap in sepsis prevention [20,36].

Addressing Socioeconomic Factors: the provision of affordable or free maternal care services is essential in regions with financial barriers [52,53,61]. Health policies in Western Europe prioritize funding for maternal health programs, while Eastern Europe often lacks similar investments due to economic constraints [3,44]. Despite subsidies for some services, hidden fees and unofficial payments can increase the financial burden and discourage women from accessing comprehensive prenatal care. Studies suggest that women in the lowest income brackets may limit their visits due to the costs associated with travel, consultations, and testing [19].

### 4.3. Importance of GBS and EOS Screening

Screening for GBS Colonization: The introduction of universal GBS screening in combination with intrapartum antibiotic prophylaxis has significantly reduced GBS-associated EOS rates in Western Europe [1,5,47,48]. This includes both culture-based and risk-based screening methods, with culture methods being more effective in detecting colonization. In contrast, GBS screening programs in Eastern Europe are limited due to resource constraints and lack of standardization [7,32].

Role of Biomarkers in EOS Detection: Biomarkers such as C-reactive protein (CRP), procalcitonin (PCT), and blood cultures are increasingly being used for EOS diagnosis in Western Europe. Their integration into clinical risk calculators helps clinicians to identify children at risk while avoiding unnecessary overuse of antibiotics [4,14]. The limited access to such diagnostic tests in Eastern Europe makes risk stratification difficult and leads to either overtreatment or overlooked cases [11].

Routine Maternal Health Screening: Antenatal health checks, including blood pressure measurement, diabetes screening, and infection testing, are essential for the detection of maternal EOS risk factors such as pre-eclampsia and chorioamnionitis [51,62]. The robust Western European maternal care frameworks emphasize these screenings, while such practices are inconsistently enforced in Eastern European regions [3,18,40,63].

Neonatal and Postpartum Surveillance: Monitoring the risk of EOS in neonates based on clinical signs and laboratory markers contributes to early detection and intervention in high-risk cases and thus to rational antibiotic use [64]. Advances in diagnostic neonatal biomarkers (e.g., procalcitonin, IL-6) and risk calculators offer promising solutions for targeted EOS management [65,66]. Combining maternal history with newborn screening is a standard practice in Western Europe but is often hampered by limited resources in Eastern Europe [12,25,67].

## 5. Conclusions

Healthcare disparities between Western and Eastern Europe highlight the urgent need for targeted investment in maternal healthcare infrastructure, comprehensive infection screening programs, and public health interventions in Eastern regions [64]. Despite Western Europe’s notable strides in EOS prevention and treatment, Eastern Europe, particularly rural areas like those in Romania, faces challenges due to inadequate healthcare infrastructure, socio-cultural barriers, and limited access to prenatal screening as well as high out-of-pocket costs and expenses continue to hinder access for vulnerable populations [9,21,26,47].

The absence of standardized surveillance systems in many Eastern European countries results in inconsistent EOS reporting [28]. In contrast, regions with rigorous epidemiologic monitoring can better assess and prevent EOS. Expanding GBS screening, improving maternal infection management, and promoting equitable health policies are essential to reducing EOS rates and improving neonatal health outcomes globally [4,23,68].

Maternal education and marital status influence antenatal care uptake, making tailored awareness campaigns for disadvantaged groups crucial. Bridging these gaps requires both health policy reforms and community-driven prevention strategies, including universal GBS screening and improved antenatal access [19,32].

This review found that half of the studies (50%) had a low overall risk of bias, indicating adherence to robust methodologies. However, 40% of studies fell into the medium-risk category, and 10% exhibited high bias risk. These findings underscore the importance of improving key areas such as sample size adequacy, transparent outcome reporting, and minimizing potential funding or conflict of interest biases. While most studies provided reliable evidence, addressing these issues is critical to improving research credibility and reproducibility.

Study heterogeneity stemmed from geographic and economic disparities, variability in study designs, and differences in population characteristics. Subgroup analyses linked higher neonatal mortality and lower prophylaxis rates to low-income settings, while EOS diagnostic criteria varied significantly. Statistical assessments revealed moderate to high heterogeneity, improved after excluding high-bias studies.

### Recommendations for Future Research

Future research should focus on adopting standardized methodologies, including robust randomization processes, blinding techniques, and comprehensive reporting of all outcomes. Studies should ensure sufficient sample sizes to improve statistical power and address potential sources of bias, such as funding and conflicts of interest, through transparent declarations. Pre-registering study protocols and adhering to open science practices, including sharing raw data, will further enhance reproducibility. Additionally, systematic reviews and meta-analyses of these studies can identify recurring methodological gaps, providing a framework for designing higher-quality studies that can reliably inform clinical or policy-related decisions.

## Figures and Tables

**Table 1:** Review findings.

Study	Type of Study	Country	Maternal Risk Factors	Socioeconomic Factors	Access to Prenatal Care
Odabasi, I. O., & Bulbul, A. (2020) [15]	Review article	Turkey (Eastern Europe and Transcontinental Region)	Premature rupture of membranes (PROM), maternal infection (chorioamnionitis), prolonged labor, and maternal GBS colonization with inadequate intrapartum antibiotic prophylaxis (IAP)	Indirect acknowledgment of healthcare inequalities affecting access to preventative measures	Limited access to prenatal care, especially in rural and lower-economic-status populations, contributes to suboptimal outcomes.
Raveh, D., et al. (2006) [16]	Observational study	General European focus, with specific references to high-income Western European settings	Maternal GBS colonization, prolonged labor, fever during delivery, and absence of IAP	Indirectly discussed; lack of standard implementation of GBS screening is linked to resource disparities in healthcare systems.	Emphasized the effectiveness of prenatal care, specifically universal maternal GBS screening.
Waters, D., et al. (2011) [8]	Systematic review	Focus on low- and middle-income countries (including some Eastern European populations)	PPROM (preterm premature rupture of membranes), chorioamnionitis, and maternal genital infections	Poverty, lack of education, and limited healthcare infrastructure contribute to delayed care and increased neonatal sepsis incidence	Poor access to maternal health services in rural areas and among socioeconomically disadvantaged groups
Barcaite, E., et al. (2008) [17]	Cross-sectional observational study	Multinational study with a broad European context	Maternal GBS colonization during late pregnancy and lack of screening or prophylactic measures	Identified as contributors to variability in the implementation of GBS screening across European countries.	Highlighted the lack of standardized GBS screening policies in several Eastern European countries, resulting in missed opportunities for IAP.
Hincu, M. A., et al. (2024) [2]	Prospective cohort study	Romania	Maternal infection (chorioamnionitis), PROM, and lack of prophylactic measures like antibiotics in the presence of identified risks	Insufficiently addressed but acknowledged in terms of accessibility to diagnostic tools	Poor, especially in rural regions, impacting screening and timely interventions
Van Herk, W., et al. (2016) [14]	Review	The Netherlands; broader implications for Europe	Prolonged rupture of membranes, fever during labor, GBS colonization, and preterm birth	Not explicitly discussed, but discrepancies in healthcare availability across countries are acknowledged	Stressed as critical to ensuring timely GBS screening and intrapartum interventions
Miteniece, E. (2021) [3]	Qualitative study	Focus on Eastern Europe (e.g., Romania, Bulgaria, Poland).	Poor maternal health outcomes indirectly lead to neonatal risks such as EOS.	Major disparities exist in access to maternal care due to poverty, healthcare system inefficiencies, and rural-urban divides.	Suboptimal access to prenatal care; many women fail to receive critical screenings during pregnancy
Panaitescu, A. M., et al. (2020) [18]	Observational study	Romania	Hypertensive disorders of pregnancy (HDP) associated with preterm birth and newborn complications.	Indirectly discussed as contributors to undiagnosed and poorly managed HDP.	Often inadequate; many women fail to receive regular prenatal visits or hypertension management
WHO. (2004) [19]	Policy analysis and review	Romania	Poor family planning, lack of access to contraception, and restrictive abortion policies leading to adverse maternal health outcomes.	Poverty and lack of access to reproductive health services are highlighted as critical issues.	Limited, compounded by insufficient family planning and poor healthcare access
Ciulpan, A., et al. (2024) [20]	Observational study	Romania	NA (Study is specifically on congenital malformations)	Limited antenatal detection attributed to socioeconomic and resource disparities	Highlighted as inadequate for timely detection of congenital heart conditions in low-income populations
Cobzeanu, M. L., et al. (2022) [21]	Observational study	Romania	History of cesarean delivery, multiparity, and uterine surgery were identified as risk factors for placenta accreta, indirectly increasing risk for neonatal complications, including prematurity that may predispose to EOS.	Limited resources and healthcare disparities in North-East Romania were noted to affect diagnosis and care.	Underscores poor access to specialized maternal care, especially in rural or underserved regions.
Chanturidze, T. (2012) [9]	Policy and technical assistance report	Romania	Identified policy gaps in maternal care indirectly leading to poor neonatal outcomes, including EOS	Widespread healthcare inequality across rural and urban regions is a key focus of this report	Poor access to essential maternal healthcare services, with specific emphasis on regional disparities in healthcare infrastructure
Hincu, M. A. (2024) [22]	Prospective cohort study	Romania	PROM, maternal chorioamnionitis, and intra-amniotic infections	Insufficient access to advanced laboratory diagnostics in low-resource regions	Limited prenatal care, particularly in rural areas, impacts maternal infection management
Kwatra, G., et al. (2016) [23]	Cross-sectional study	Multinational, emphasizing global prevalence, with inclusion of European countries	Maternal GBS colonization and inadequate IAP implementation	Lack of universal screening programs, particularly in resource-constrained countries	GBS screening inconsistencies impact maternal and neonatal care, especially in under-resourced region
Melin, P. (2011) [24]	Review	General focus on Europe and North America	GBS colonization, PROM, fever during labor, and lack of IAP	Inconsistent implementation of universal screening programs in lower-resource countries impacts neonatal outcomes	Limited access to routine prenatal GBS screening noted in low-resource regions
Stocker, M. (2016) [14]	Review	Europe (general focus)	PROM, GBS colonization, and chorioamnionitis	Not addressed directly, but disparities in healthcare are acknowledged as impacting neonatal outcomes	Advocates for consistent GBS screening and maternal care
Johansson Gudjónsdóttir, M., et al. (2019) [25]	Observational study	Sweden	Chorioamnionitis and maternal GBS colonization	NA (Not addressed)	Emphasis on universal healthcare enabling comprehensive screening
Schrag, S. J., et al. (2016) [1]	Systematic review and meta-analysis	Europe (includes multiple countries with comparisons between Western and Eastern European nations)	Maternal GBS colonization, PROM, and lack of intrapartum antibiotic prophylaxis (IAP)	Variability in screening and prophylaxis policies linked to economic disparities	Limited in resource-constrained areas, especially in Eastern Europe, affects GBS prevention
Oikonomou, I., et al. (2020) [11]	Observational multicenter cohort study	Europe (multiple participating countries, including both Western and Eastern Europe)	PROM, chorioamnionitis, maternal fever, and GBS colonization	Disparities noted between countries in maternal care and access to standardized screening	Identified lack of prenatal care in low-income settings as a contributor to missed risk factor
Giannoni, E., et al. (2018) [4]	Cross-sectional observational study	Europe (data from neonatal units across both Western and Eastern regions)	Intrapartum infections, PROM, and prolonged labor	Overuse of broad-spectrum antibiotics and limited resources in Eastern Europe contribute to resistance patterns	Varied; inconsistencies in maternal care access exacerbate infection risks in certain regions
Almeida, A., et al. (2019) [26]	Economic analysis and qualitative research	Eastern Europe (focus on Romania, Poland, and Bulgaria)	Related to inadequate or delayed prenatal care, leading to increased maternal infections and preterm births	High out-of-pocket healthcare costs prevent access to regular prenatal care	Cost as a predominant barrier, disproportionately affecting rural and low-income populations
Gkentzi, D., et al. (2021) [13]	Review article	Europe	Indirectly discussed; linked to maternal infections and lack of maternal screening programs	Limited availability of advanced diagnostics like procalcitonin measurements in low-resource settings	Not explicitly discussed
Berardi, A., et al. (2017) [27]	Review of guidelines and practice	Europe (comparison of national practices across countries)	GBS colonization, PROM, maternal fever, and preterm birth	Limited resources and inconsistencies in practice standards between countries result in increased risks in underserved areas	Advocates for universal prenatal GBS screening programs to ensure timely prophylaxis
Flidel-Rimon, O., et al. (2022) [10]	Review article	Global focus, including Europe	Indirectly related to risk factors promoting neonatal infections (PROM, maternal infections, and prolonged labor)	Inefficient infection prevention due to resource constraints and overuse of broad-spectrum antibiotics in low-resource settings	Not discussed
Mukhopadhyay, S., et al. (2019) [12]	Multicenter observational study	Europe	Maternal fever, PROM, GBS colonization, and prolonged labor	Barriers to implementation in low-resource settings include lack of access to real-time maternal and neonatal data	Inadequate care in certain regions delays the effectiveness of EOS prevention tools
Hansen, N. I., et al. (2016) [28]	Cross-sectional study	Europe (country-level analysis)	GBS colonization, prolonged labor, maternal fever	Healthcare disparities exacerbate variability in EOS incidence and prevention	Identified inconsistencies in GBS screening contribute to variable EOS rates.

**Table 2:** Incidence of EOS and diagnostic approaches.

Study	Incidence	Laboratory Findings	Treatment Protocols
Odabasi, I. O., & Bulbul, A. (2020) [15]	Estimated 0.5–2 cases per 1000 live births in developed regions, potential higher rates in areas with inadequate maternal-infant healthcare systems	Common biomarkers include CRP (C-reactive protein), procalcitonin, and blood culture results, emphasizing timely diagnosis	Standard empiric antibiotic regimen includes ampicillin and gentamicin. Resistance to these therapies was recognized as a growing challenge
Raveh, D., et al. (2006) [16]	1–3 cases per 1000 live births in high-income countries; under-reported in resource-limited settings without universal screening	Positive cultures are used for diagnosing GBS infection in EOS cases.	Routine use of IAP with penicillin/amoxicillin for GBS-positive mothers; consideration of alternative antibiotics for penicillin-allergic patients.
Waters, D. [8]	Much higher in low-resource settings, with rates of 4–6 per 1000 live births reported in some regions	Diagnostics such as blood cultures and complete blood counts are inconsistently available in resource-constrained settings	Empiric antibiotic therapy (typically ampicillin and gentamicin) is standard; however, delays in initiating treatment due to diagnostic constraints are common.
Barcaite, E., et al. (2008) [17]	GBS-related EOS risk is strongly linked to maternal colonization incidence: 0.5–2 cases per 1000 live births depending on screening practices	NA (Study does not focus on newborn diagnostics).	Advocates for universal GBS screening and IAP to mitigate EOS rates.
Hincu, M. A., et al. (2024) [2]	NA (Rates of EOS not specified)	Procalcitonin and CRP levels were explored as biomarkers for early laboratory confirmation of EOS. Additionally, elevated interleukin-6 levels were noted	Empiric antibiotic therapy with ampicillin and gentamicin is standard but tailored as per local resistance patterns
Van Herk, W., et al. (2016) [14]	0.8–1.2 cases per 1000 live births in high-income settings with effective prevention measures.	Elevated procalcitonin and CRP levels identified as useful diagnostic indicators; culture confirmation is emphasized.	Advocated for judicious empiric antibiotic use (ampicillin and gentamicin) to prevent resistance.
Hincu, M. A. (2024) [22]	NA (Study focuses on diagnostics and biomarkers)	Elevated procalcitonin, CRP, and neutrophil-to-lymphocyte ratio were identified as diagnostic markers for EOS	Empiric antibiotic protocols are discussed, including ampicillin and gentamicin as first-line treatment
Kwatra, G., et al. (2016) [23]	GBS-related EOS incidence ranges from 0.5–2 per 1000 live births in screened populations and higher in unscreened regions	NA (Focus on maternal colonization, not newborn diagnostics)	Emphasis on GBS screening and IAP in reducing neonatal GBS-related sepsis
Melin, P. (2011) [24]	Incidence in high-resource regions ranges from 0.4–1.3 per 1000 live births	Sepsis diagnosis depends on culture positivity for GBS in neonates	Advocates for universal screening and IAP with penicillin for GBS-positive mothers to prevent EOS
Rosychuk, R. J. (2014) [29]	NA (Focus on diagnostic value of CRP)	CRP measurement at 18 h post-birth was found to have high negative predictive value for ruling out EOS	Supports reducing unnecessary antibiotic use based on CRP findings
Stocker, M. (2016) [14]	0.8–1.2 cases per 1000 live births	Biomarkers such as CRP and PCT combined with clinical presentation are emphasized	Empiric antibiotics (ampicillin and gentamicin) widely recommended
Johansson Gudjónsdóttir, M., et al. (2019) [25]	Overall EOS incidence: 0.6–1.3 per 1000 live births	Blood cultures remain the gold standard; CRP and PCT are used as adjuncts	Penicillin recommended for GBS prophylaxis; empiric AMP-GENT for neonatal treatment
Schrag, S. J., et al. (2016) [1]	0.4–2.1 cases per 1000 live births, with higher rates reported in areas without universal GBS screening	Positive blood cultures for GBS were used to confirm diagnosis	Universal GBS screening and IAP with penicillin are recommended to reduce EOS risk
Giannoni, E., et al. (2018) [4]	0.6–2.8 cases per 1000 live births; higher in Eastern Europe, partly due to antibiotic resistance	Blood cultures identified resistant pathogens such as multidrug-resistant gram-negative bacteria	Advocates for antimicrobial stewardship programs and narrower-spectrum empiric antibiotic regimens
Gkentzi, D., et al. (2021) [13]	NA	Procalcitonin is highlighted as a highly specific and early biomarker for EOS, with predictive accuracy	Early empiric antibiotic therapy may be modified based on procalcitonin trends
Berardi, A., et al. (2017) [27]	Lower in countries with universal GBS screening, ~0.3–0.8 cases per 1000 live births	Not emphasized (focus is on maternal screening and prevention)	NA (recommends IAP with penicillin or alternatives for GBS carriers)
Flidel-Rimon, O., et al. (2022) [10]	NA (Focus on antibiotic resistance trends rather than incidence rates)	Highlights challenges in detecting resistant pathogens in blood cultures	Urges reduced overuse of broad-spectrum antibiotics; focuses on tailored antimicrobial use
Mukhopadhyay, S., et al. (2019) [12]	NA (Focus on risk stratification tools rather than incidence rates)	Risk tools rely on combining clinical and laboratory data such as CRP and blood culture	Risk calculators reduce unnecessary antibiotic use; empiric regimes adjusted based on risk assessment
Hansen, N. I., et al. (2016) [28]	Varies by country, ranging from 0.4–2.5 cases per 1000 live births	Diagnostic tools vary, with blood culture as the main method	Variations in empiric antibiotic use between countries due to differences in pathogen prevalence

Not available (NA).

## Data Availability

Data supporting the reported results can be found at mirelasiminel@gmail.com, anca.vulcanescu@umfcv.ro.

## Supplementary Materials

The following supporting information can be downloaded at: https://www.mdpi.com/article/10.3390/life15020292/s1.
