# Correction: Vulcănescu et al. Systematic Review: Maternal Risk Factors, Socioeconomic Influences, Neonatal Biomarkers and Management of Early-Onset Sepsis in Late Preterm and Term Newborns—A Focus on European and Eastern European Contexts. *Life* 2025, *15*, 292

**DOI:** 10.3390/life15040616

**Published:** 2025-04-07

**Authors:** Anca Vulcănescu, Mirela-Anișoara Siminel, Sorin-Nicolae Dinescu, Mihail-Virgil Boldeanu, Anda-Lorena Dijmărescu, Maria-Magdalena Manolea, Constantin-Cristian Văduva

**Affiliations:** 1 “Filantropia” Clinical Municipal Hospital, 200143 Craiova, Romania; anca.vulcanescu@umfcv.ro (A.V.); mihail.boldeanu@umfcv.ro (M.-V.B.); lorena.dijmarescu@umfcv.ro (A.-L.D.); magdalena.manolea@umfcv.ro (M.-M.M.); cristian.vaduva@umfcv.ro (C.-C.V.); 2 University of Medicine and Pharmacy of Craiova, 200349 Craiova, Romania; 3 Department of Neonatology, University of Medicine and Pharmacy of Craiova, 200349 Craiova, Romania; 4 Department of Epidemiology, University of Medicine and Pharmacy of Craiova, 200349 Craiova, Romania; sorin.dinescu@umfcv.ro; 5 Clinical Emergency County Hospital, 200642 Craiova, Romania; 6 Department of Immunology, University of Medicine and Pharmacy of Craiova, 200349 Craiova, Romania; 7 Department of Obstetrics and Gynecology, University of Medicine and Pharmacy of Craiova, 200349 Craiova, Romania

## Corresponding Author Corrected

In the original publication [1] there was an error regarding the corresponding authors Anca Vulcănescu and Mirela-Anișoara Siminel. The corrected sole Corresponding author appears here: Mirela-Anișoara Siminel. The authors state that the scientific conclusions are unaffected. This correction was approved by the Academic Editor. The original publication has also been updated.
