# Supplementary material for: Systematic Review: Maternal Risk Factors, Socioeconomic Influences, Neonatal Biomarkers and Management of Early-Onset Sepsis in Late Preterm and Term Newborns—A Focus on European and Eastern European Contexts
Source: Life (Basel). 2025 Feb 13;15(2):292. doi: 10.3390/life15020292 (PMC11856718; doi:10.3390/life15020292)
Supplement: Supplementary file 1 [file life-15-00292-s001.zip › life-3454497-supplementary.pdf]

Supplementary Material S1. **Search Strategy (expanded)**

1. Databases Used

1. **PubMed**
2. **Google Scholar**
3. **ScienceDirect**
4. **Scopus**

2. Search String Components

The search string was constructed using Boolean operators (AND, OR, NOT), field-specific search operators, and controlled vocabulary (e.g., MeSH terms for PubMed).

3. Search Strings by Database

PubMed

Search String: ("early-onset sepsis"[Title/Abstract] OR "EOS"[Title/Abstract] OR "neonatal sepsis"[MeSH Terms] OR "neonatal infections"[Title/Abstract]) AND ("maternal healthcare"[MeSH Terms] OR "antenatal care"[MeSH Terms] OR "maternal risk factors"[Title/Abstract]) AND ("Europe"[MeSH Terms] OR "Eastern Europe"[Title/Abstract] OR "Romania"[Title/Abstract] OR "socioeconomic factors"[MeSH Terms]) AND ("Group B Streptococcus"[MeSH Terms] OR "GBS"[Title/Abstract]) NOT ("animal studies"[MeSH Terms])

Filters Applied:

- Language: English
- Publication Date: January 2004 – April 2024
- Study Types: Peer-reviewed articles, systematic reviews, observational studies

Google Scholar

Search String: "early-onset sepsis" OR "neonatal sepsis" OR "EOS" AND ("maternal healthcare" OR "antenatal care" OR "maternal risk factors") AND ("Europe" OR "Eastern Europe" OR "Romania") AND ("Group B Streptococcus" OR "GBS")

Advanced Filters Applied:

- Include only articles published between 2004 and 2024.
- Include only articles in English.
- Exclude patents and citations.

ScienceDirect

Search String: TITLE-ABSTR-KEY("early-onset sepsis" OR "neonatal sepsis") AND TITLE-ABSTR KEY("maternal healthcare" OR "antenatal care" OR "maternal risk factors") AND TITLE-ABSTR-KEY("Europe" OR "Eastern Europe" OR "Romania") AND TITLE-ABSTR-KEY("Group B Streptococcus" OR "GBS")

Filters Applied:

- Year: 2004–2024
- Document Type: Research articles, reviews
- Language: English

Scopus

Search String: TITLE-ABS-KEY("early-onset sepsis" OR "neonatal infections" OR "EOS") AND TITLE-ABS-KEY("maternal risk factors" OR "antenatal care" OR "maternal healthcare") AND TITLE-ABS-KEY("Europe" OR "Eastern Europe" OR "Romania") AND TITLE-ABS-KEY("Group B Streptococcus" OR "GBS")

Filters Applied:

- Year: 2004–2024
- Language: English
- Source Type: Articles, reviews

Supplementary Material S2. **Detailed Risk of Bias Assessment**

Study	Study Design Bias	Sample Size Bias	Outcome Reporting Bias	Funding Bias	Conflict of Interest Bias
Odabasi, I. O., & Bulbul, A. (2020)	Low	Low	Low	Low	Low
Raveh, D., et al.	Medium	Medium	Medium	Medium	Medium
Waters, D., et al	Low	Low	Low	Low	Low
Barcaite, E., et al. (2008)	Low	High	Low	Low	Low
Hincu, M. A., et al. (2024)	Medium	Medium	Medium	Medium	Medium
Van Herk, W., et al. (2016)	Low	Low	Low	Low	Low
Miteniece, E. (2021)	Medium	Medium	Medium	Medium	Medium
Panaitescu, A. M., et al. (2020)	Medium	Medium	Medium	Medium	Medium
WHO. (2004)	Low	Low	Low	Low	Low
Ciulpan, A., et al. (2024)	High	High	High	High	High
Cobzeanu, M. L., et al. (2022)	Medium	Medium	Medium	Medium	Medium
Chanturidze, T. (2012)	Low	Medium	Medium	Medium	Medium
Hincu, M. A. (2024)	High	High	High	High	High
Kwatra, G., et al. (2016)	Low	Low	Low	Low	Low
Melin, P. (2011)	Medium	Low	Medium	Medium	Medium
Stocker, M. (2016)	Low	Low	Low	Low	Low
Johansson Gudjónsdóttir, M., et al. (2019)	Low	Low	Low	Low	Low
Schrag, S. J., et al. (2016)	Medium	Low	Medium	Medium	Medium
Oikonomou, I., et al. (2020)	Low	Low	Low	Low	Low
Giannoni, E., et al. (2018)	Low	Low	Low	Low	Low
Almeida, A., et al. (2019)	Low	Medium	Low	Low	Low
Gkentzi, D., et al. (2021)	Medium	Low	Medium	Medium	Medium
Berardi, A., et al. (2017)	Low	Low	Low	Low	Low
Flidel-Rimon, O., et al. (2022)	Medium	Medium	Medium	Medium	Medium
Mukhopadhyay, S., et al. (2019)	Low	Low	Low	Low	Low
Hansen, N. I., et al. (2016)	Low	Low	Low	Low	Low
WHO, UNICEF (2020)	Low	Low	Low	Low	Low
Barcaite, E., et al. (2008)	Medium	Medium	Medium	Medium	Medium
Hincu, M. A., et al. (2024)	Medium	Medium	Medium	Medium	Medium

1. Study Design Bias: Refers to methodological flaws in how the study was structured. Rigorous study designs minimized bias, while studies with unclear protocols or control measures were rated higher.

- **Low Risk:** 50% of studies (e.g., Waters, D., et al.; Odabasi, I. O., & Bulbul, A. (2020))
- **Medium Risk:** 40% of studies (e.g., Raveh, D., et al.; Hincu, M. A., et al. (2024))
- **High Risk:** 10% of studies (e.g., Ciulpan, A., et al. (2024))

2. Sample Size Bias: Bias introduced when sample sizes are too small to detect meaningful effects. Studies with adequate power and larger populations were at lower risk.

- **Low Risk:** 50% of studies (e.g., WHO. (2004); Waters, D., et al.)
- **Medium Risk:** 35% of studies (e.g., Panaitescu, A. M., et al.; Gkentzi, D., et al. (2021))
- **High Risk:** 15% of studies (e.g., Barcaite, E., et al.; Ciulpan, A., et al.)

3. Outcome Reporting Bias: Occurs when reported outcomes are selective or incomplete. Transparent outcome reporting and pre-registered protocols lowered risk.

- **Low Risk:** 55% of studies (e.g., Schrag, S. J., et al.; Stocker, M. (2016))
- **Medium Risk:** 35% of studies (e.g., Hincu, M. A., et al.; Gkentzi, D., et al.)
- **High Risk:** 10% of studies (e.g., Cobzeanu, M. L., et al.)

4. Funding Bias: Bias introduced by funding sources influencing results. Studies with independent funding or clear declarations of financial interests had lower risks.

- **Low Risk:** 55% of studies (e.g., Odabasi, I. O., & Bulbul, A.; Johansson Gudjónsdóttir, M., et al.)
- **Medium Risk:** 35% of studies (e.g., Chanturidze, T.; Hincu, M. A., et al.)
- **High Risk:** 10% of studies (e.g., Ciulpan, A., et al.)

5. Conflict of Interest Bias: Occurs when financial or personal interests may influence study outcomes. Clear disclosures and lack of vested interests mitigated conflict of interest bias.

- **Low Risk:** 55% of studies (e.g., Mukhopadhyay, S., et al.; Berardi, A., et al.)
- **Medium Risk:** 35% of studies (e.g., Almeida, A., et al.; Gkentzi, D., et al.)
- **High Risk:** 10% of studies (e.g., Ciulpan, A., et al.)

Conclusion

The review reveals that **50% of studies exhibited a low overall risk of bias**, while **40% were categorized as medium risk**, and **10% showed high risk**. These findings highlight that most studies adhered to robust methodologies but encountered weaknesses in sample size, reporting transparency, and funding disclosures. Future research should prioritize adequate sample sizes, rigorous designs, and full reporting to reduce potential biases. Addressing medium and high-risk factors will further enhance the validity and reproducibility of study findings.
